# Non-tumor-related prognostic factors for immunotherapy–chemotherapy or immunotherapy alone as first-line in advanced non-small cell lung cancer (NSCLC)

**DOI:** 10.1007/s10238-024-01298-z

**Published:** 2024-03-15

**Authors:** Ziwei Guo, Xing Wei, Chuanhao Tang, Jun Liang

**Affiliations:** Department of Oncology, Peking University International Hospital, Beijing, 102206 China

**Keywords:** Non-small cell lung cancer, Immunotherapy, Biomarkers, Prognosis

## Abstract

Besides programmed death ligand 1 (PD-L1) expression, rapid, cost-effective and validated scores or models are critical for the prognosis and prediction of patients received immune checkpoint inhibitors (ICIs). In this retrospective study, 182 patients with NSCLC receiving ICIs from 2015 to 2022 were divided 1:1 into a training cohort and a validation cohort. We identified a score established by three factors and analyzed the prognostic implications by Kaplan–Meier approach (Log rank test) and time-dependent receiver operating characteristic (ROC) analyses. A non-tumor-related score (NTRS) was established that could be used as a prognostic factor (HR 2.260, 95% CI 1.559–3.276, *P* < 0.001 in training cohort; HR 2.114, 95% CI 1.493–2.994, *P* < 0.001 in validation cohort) and had a high time-dependent ROC for overall survival (OS) (AUC 0.670–0.782 in training cohort; AUC 0.682–0.841 in validation cohort). PD-L1 (1–49%) and NTRS (score = 0, 1, 2, 3) combination significantly improved the assessment of patients’ OS and progress-free survival (PFS), which was statistically different in training cohorts (*P* < 0.001 for OS, 0.012 for PFS) and validation cohorts (*P* = 0.01 for OS, < 0.001 for PFS). The NTRS provided a better assessment of durable clinical benefit (DCB) compared to PD-L1 expression (*P* = 0.009 vs. 0.232 in training cohort; *P* = 0.004 vs. 0.434 in validation cohort). NTRS may help improve prognosis stratification of patients receiving ICIs in first-line NSCLC and may be combined with tumor-related parameters.

**Supplementary Information:**

The online version contains supplementary material available at 10.1007/s10238-024-01298-z.

## Introduction

ICIs have revolutionized the treatment landscape of NSCLC. ICIs have been approved for first-line therapy and are available either alone (PD-L1 tumor percentage score (TPS) ≧ 50%) or in combination with chemotherapy [1, 2]. Several prognostic and predictive factors in NSCLC have been described in association with ICIs. Some clinical studies have shown that PD-L1, tumor mutational burden (TMB), microsatellite instability (MSI), defective mismatch repair (dMMR) and other immune features are considered as potential biomarkers for predicting ICIs [3–6]. However, only high levels of MSI/dMMR and TMB (no universal definition of high TMB; high cost; limited by tissue sample size) are expressed in a minority of patients [3, 7–9]. Despite Keynote-024 [10] and Keynote-042 [11] demonstrated a benefit in OS in patients receiving ICIs with PD-L1 ≧ 50%, patients in Keynote-189 [12] and 407 [13] with ICIs combined with chemotherapy, regardless of PD-L1, had a significantly superior objective response rate (ORR), PFS, and OS. Therefore, the predictive and prognostic value of PD-L1 expression is limited.

Despite the tremendous progress of ICIs in the treatment of NSCLC, there are still many outstanding issues in patient prognosis and efficacy that need to be solved. Some studies showed genetic models [14, 15], neural network model [16], and nutrition index [17] could predict the risk of NSCLC progression. However, many models had not reached clinical practice. Obviously, this was related to economic and methodological complexity and/or tumor heterogeneity. There is growing knowledge about the importance of comprehensive prediction as a biomarker or model. Clinically relevant prognostic models are also being performed in full speed. Appropriate clinical models in early NSCLC with adjuvant chemotherapy [18], advanced NSCLC in old age [19] and lymph node metastases (LNMs) [17] have been studied as prognostic models. Nevertheless, non-tumor-related predictive and prognostic models without specific population restrictions in advanced NSCLC patients receiving ICIs are controversial. Worse yet, the relative importance of prognostic factors in the era of ICIs is currently not well defined and explored. In the clinical field, there is an urgent need for valid, simple, cost-effective, and comprehensive model predictions to identify patients who may benefit from ICIs.

This study aimed to explore potential model and to accurately predict prognosis in patients with advanced NSCLC. A new NTRS could more precisely assess the predictive and prognostic value of NSCLC patients undergoing ICIs and more clearly identify the relative prognostic impact of clinical factors. The NTRS established a new approach that can be applied to the prediction and prognosis of NSCLC.

## Materials and methods

This retrospective study was approved by the Research Ethic Committee of Peking University International Hospital’s, and all patients provided their informed consent to treatment including immunotherapy. The process followed the declaration of Helsinki.

### Study population

We retrospectively screened 1796 NSCLC patients at XX Hospital from Jan. 2015 to Jun. 2022. All patients diagnosed with histologically confirmed stage IV or unresectable and non-curative stage III NSCLC who received at least two doses of first-line therapy were enrolled. Inclusion criteria were histologically or cytologically confirmed NSCLC with at least one measurable tumor lesion according to response evaluation criteria in solid tumors (RECIST) version 1.1, adequate bone marrow and organ function, and an expected survival time of ≧ 3 months. Exclusion criteria were patients with tumors with targetable mutations (e.g., EGFR mutations, ROS1 and ALK translocations), patients enrolled in clinical trials, patients receiving antitumor therapy such as chemotherapy, radiation, targeted and immunotherapy prior to the first dose, use of systemic glucocorticoids 4 weeks prior to the first dose, and metabolic syndrome such as type 2 diabetes, hypertension, family history of obesity, atherosclerosis, fatty liver and BMI > 27. Due to patient blood tests resulted after diagnosis, patients were placed on a relatively standardized diet with a reference daily sugar, protein and lipid ratio of 3:2:1. Enrollment data included patient characteristics (age, gender, smoking history, Eastern Cooperative Oncology Group (ECOG) status, blood tests, biochemical tests, coagulation tests and tumor marker tests), tumor characteristics (histology, stage, metastatic sites, and PD-L1 expression, baseline tumor size (BTS), best overall response (BOR), and DCB).

### Blood sample analysis

Blood tests performed within 1 month prior to treatment were involved in the analysis, and laboratory blood tests were performed. The SYSMEX XN3000 analyzer was used to determine the levels of routine blood (white blood cells, lymphocytes, neutrophils, platelets and hematocrit (HCT)). The BECKMAN AU5800 automatic biochemical analyzer measured lactate dehydrogenase (LDH) (the upper limit of normal (ULN) of 250 U/L), alkaline phosphatase (ALP) (ULN of 125 U/L), albumin (ALB) (the lower limit of normal (LLN) of 40 g/L), high-density lipoprotein cholesterol (HDL-C) (LLN of 1.0 mmol/L), low-density lipoprotein cholesterol (LDL-C) (ULN of 3.4 mmol/L), free fatty acids (ULN of 769 μEq/L), and β2-microglobulin (ULN of 2.64 mg/L). ACL-TOP 700 automatic coagulation analyzer was used to test plasma fibrinogen (FIB) (ULN of 400 ng/dL). CobasE601 analyzer examined carcinoembryonic antigen (CEA) (ULN of 5 ng/mL), squamous epithelial cell carcinoma-associated antigens (SCC) (ULN of 1.5 ng/mL), and CYFRA21-1 (ULN of 3.3 ng/mL). Each patient underwent baseline chest and abdominopelvic computed tomography (CT) scans as well as magnetic resonance imaging (MRI) of the brain and bone scans as necessary less than 8 weeks prior to the first ICI.

### PD-L1 staining

PD-L1 staining was obtained from pathology reports or results from genetic testing. PD-L1 staining was used immunohistochemistry by the 22C3 PharmDx assay (Dako North America). PD-L1 expression was evaluated according to the percentage of membranous stained tumor cells, also known as TPS, where PD-L1 < 1%, 1% ≦ PD-L1 < 50% and PD-L1 ≧ 50% [20–22].

### Scoring model development and validation

They were randomized splitting into training and validation groups and then stratified by gender and age. NTRS was developed in the training cohort. To determine the prognostic significance of each factor in the baseline clinical variables (age, gender, smoking history, pathology type, ECOG status, CEA, SCC, CYFRA21-1, size, T-stage, N-stage, metastatic sites, and PD-L1 expression), the best cutoff values were screened and validated to confirm the prognostic ability. Three significant factors were eventually included in the score. One point was scored for each of these three variables, and the total score was defined as the scoring model. The discriminative ability of the model was assessed by the area under the curve (AUC). The accuracy of the model was determined by the Harrell’s Discriminant Consistency Index (C-index).

### Efficacy assessment and treatment modalities

These measurable lesions were performed according to RECIST. Based on National Comprehensive Cancer Network (NCCN) guideline recommendations and PD-L1 expression, patients were treated with single-agent immunization or immune-chemotherapy treatment. In addition, patients with ECOG≧ 2 partially received immune-monotherapy according to family wishes.

### Follow-up

Patients were followed up primarily by outpatient visits (or telephone) every 10 ± 2 weeks. PFS was defined as the time from initiation of treatment to disease progression or death. OS was defined as the time from initiation of treatment to death. DCB was determined as a complete response (CR), partial response (PR) or stable disease (SD) lasting longer than 6 months; no durable benefit (NDB) was characterized as progressed disease (PD) or SD lasting less than 6 months. BOR was measured as CR/SD/PR groups. Patients with no events were censored at the last follow-up. Missing data rates were < 15% to 20%.

### Statistical analysis

IBM SPSS (version 26.0; SPSS Inc, Chicago, IL, USA), MedCalc (version 20.1.0) and R software (http://www.r-project.org/) version 3.6.1 were used to conduct statistical analysis. Continuous variables were presented by median with interquartile ranges (IQRs) and categorical variables were expressed as frequencies (%). Continuous and categorical variables were assessed by two-side Mann–Whitney *U* test and chi-square test or Fisher’s exact test, respectively. Kaplan–Meier method was used to estimate PFS and OS, and differences were compared using the log-rank test. X-tile [23] analysis was determined as an identification of the optimal cutoff for 5-year OS with factors including neutrophil, lymphocyte, high neutrophil lymphocyte ratio (NLR), platelets, platelets lymphocyte ratio (PLR), HCT, β2-microglobulin, FIB, LDH, ALB, HDL-C, ALP, CYFRA21-1, SCC, CEA. Univariable and multivariable Cox regression analyses were performed to determine independent factors of OS or PFS by the Cox regression methods. Time-dependent ROC curve analyses used the “timeROC” package to evaluate the prognostic and predictive ability of each factor. *P* values < 0.05 were considered statistically different.

## Results

### Patients clinicopathologic characteristics

Clinicopathological analysis was performed on 182 patients receiving immunotherapy combined with platinum-based chemotherapy or monotherapy according to PD-L1 TPS or ECOG status. These patients were randomized in a 1:1 ratio into a training cohort and a validation cohort (Table 1). The median follow-up for the training cohort was 34.6 months, with a median OS of 26.3 months (95% confidence interval (CI) 18.5–34.1); the 1-, 3-, and 5-year OS rates were 78%, 33%, and 12%, respectively. The median PFS was 7.6 months (95% CI 5.9–9.4). The median follow-up for the validation cohort was 34.2 months, with a median OS of 32.5 months (95% CI 25.5–39.5); the 1-, 3-, and 5-year OS rates were 88%, 44%, and 3%, respectively. The median PFS was 7.7 months (95% CI 6.5–8.8). In summary, the demographics, tumor characteristics and treatment regimens were highly comparable in the two cohorts, except for the higher proportion of patients with PD-L1≧ 1% in the training cohort (80.2% vs. 64.8%) compared to the validation cohort.

**Table 1:** Baseline characteristics of patients

Factors	All patients (*n* = 182)	Training (*n* = 91)	Validation (*n* = 91)	*p*
	Patients no. (%)			
Age (median IQR), y	66 (62–71)	65 (61–70)	66 (62–70)	0.40
*Sex*				0.87
Male	130 (71.4)	66 (72.5)	64 (70.3)	
Female	52 (28.6)	25 (27.5)	27 (29.7)	
*Smoking*				0.36
Yes	116 (63.7)	61 (67)	55 (60.4)	
No	66 (36.3)	30 (33)	36 (39.6)	
*ECOG*				0.51
0–1	157 (86.3)	77 (84.6)	80 (87.9)	
> 2	24 (13.2)	14 (15.4)	10 (11.0)	
Missing data	1 (0.5)	0	1 (1.1)	
*Pre-treatment CEA, ng/mL*				0.88
> 5	85 (46.7)	43 (47.3)	42 (46.2)	
≦ 5	97 (53.3)	48 (52.7)	49 (53.8)	
*Pre-treatment CYFRA21-1, ng/mL*				0.75
> 3.3	126 (69.2)	62 (68.1)	64 (70.3)	
≦ 3.3	56 (30.8)	29 (31.9)	27 (29.7)	
*Pre-treatment SCC, ng/mL*				0.31
> 1.5	63 (34.6)	27 (29.7)	36 (39.6)	
≦ 1.5	119 (65.4)	64 (70.3)	55 (60.4)	
*Pre-treatment LDH, U/L*				0.25
> ULN	53 (29.1)	23 (25.3)	30 (33.0)	
≦ ULN	129 (70.9)	68 (74.7)	61 (67.0)	
*Pre-treatment ALP, U/L*				0.86
> ULN	41 (22.5)	20 (22.0)	21 (23.0)	
≦ ULN	141 (77.5)	71 (78.0)	70 (77.0)	
*Pre-treatment β2-microglobulin, mg/L (normal creatinine levels)*				0.44
> ULN	32 (17.6)	14 (15.4)	18 (19.8)	
≦ ULN	150 (82.4)	77 (84.6)	73 (80.2)	
*Pathology*				0.56
LUAD	103 (56.6)	55 (60.4)	48 (52.7)	
LUSC	74 (40.7)	34 (37.4)	40 (43.9)	
Others	5 (2.7)	2 (2.2)	3 (3.3)	
*T_staging*				0.69
*T*1	15 (8.2)	7 (7.7)	8 (8.8)	
*T*2	41 (22.5)	19 (20.9)	22 (24.2)	
*T*3	23 (12.7)	11 (12.1)	12 (13.2)	
*T*4	52 (28.6)	24 (26.4)	28 (30.7)	
Missing data	51 (28.0)	30 (32.9)	21 (23.1)	
*N_staging*				0.21
*N*0	12 (6.6)	3 (3.3)	9 (9.9)	
*N*1	16 (8.8)	7 (7.7)	9 (9.9)	
*N*2	56 (30.8)	26 (28.5)	30 (33.0)	
*N*3	75 (41.2)	44 (48.4)	31 (34.0)	
Missing data	23 (12.6)	11 (12.1)	12 (13.2)	
*M_staging*				0.47
M0	24 (13.2)	13 (14.3)	11 (12.1)	
M1	156 (85.7)	76 (83.5)	80 (87.9)	
Missing data	2 (1.1)	2 (2.2)	0	
*BTS, mm*				0.81
≦ 86	128 (70.3)	65 (71.4)	63 (69.2)	
> 86	51 (28.0)	24 (26.4)	27 (29.7)	
Missing data	3 (1.7)	2 (2.2)	1 (1.1)	
*Number of metastases*				0.28
0	27 (14.8)	14 (15.4)	13 (14.3)	
1–2	114 (62.6)	61 (67.0)	53 (58.2)	
> 2	41 (22.6)	16 (17.6)	25 (27.5)	
*PD-L1 TPS*				0.01
< 1%	50 (27.5)	16 (17.6)	34 (37.4)	
1–49%	82 (45.1)	44 (48.4)	38 (41.8)	
≧ 50%	50 (27.4)	31 (34.0)	19 (20.8)	
*Treatment patterns hasis*				
Immuno.-chemo	121 (66.5)	54 (59.3)	67 (73.6)	0.06
ICIs alone	61 (33.5)	37 (40.7)	24 (26.4)	

CI, confidence interval; OS, overall survival; PFS, progression-free survival; IQR, interquartile range; ECOG, Eastern Cooperative Oncology Group; CEA, carcinoembryonic antigen; SCC, squamous epithelial cell carcinoma-associated antigens; LDH, lactate dehydrogenase; ULN, upper limit of normal; ALP, alkaline phosphatase; LUAD, lung adenocarcinoma; LUSC, lung squamous cell carcinoma; BTS, baseline tumor size; PD-L1, programmed cell death ligand 1; TPS, tumor cell proportion score

### Establishment of the NTRS

X-tile software was used to identify the best cutoff values for 15 indicators, including neutrophils, lymphocyte, platelets, NLR, PLR, HCT, β2-microglobulin, ALB levels, ALP levels, FIB levels, LDH levels, HDL-C levels, and CYFRA21-1 levels, SCC, and CEA. The results suggested that in the training cohort, the optimal cutoff values for ALP, HDL-C, and CYFRA21-1 levels were 162 U/L, 0.7 mmol/l and 6.3 ng/ml, respectively (Fig. S1A, B, C). Thus, three independent predictors of poor OS, namely a pooled cohort with ALP, HDL-C, and CYFRA21-1 were included in NTRS (Fig. S2). One point was scored for each factor and the total score (0–3) was calculated. For patients distinguished by the best cutoff value for the other indicators, there was no distinction between the groups with high and low best cutoff values in terms of OS or *P*-values that were statistically significant.

### Identification of the NTRS with OS in the training and validation cohort

Univariate and multivariate Cox regression analyses in Table 2 and Table S1 demonstrated the relationship between clinicopathological variables and OS and PFS in the training and validation cohorts. In the univariate analysis of the training and validation cohorts, PD-L1 expression, maximum diameter, LDH levels, CYFRA21-1 levels and NTRS were associated with OS; NTRS was associated with PFS. These variables were subsequently included in multivariate analyses.

**Table 2:** Univariate and multivariate analyses for predictors of overall survival and progress-free survival in the training cohort

Variables	Overall survival				Progress-free survival			
	Univariate analysis		Multivariate analysis		Univariate analysis		Multivariate analysis	
	HR (95% CI)	*p*	HR (95% CI)	*p*	HR (95% CI)	*p*	HR (95% CI)	*p*
Age	1.004 (0.968–1.041)	0.842			1.025 (0.996–1.054)	0.095		
Male	1.255 (0.416–1.528)	0.494			0.797 (0.531–1.551)	0.494		
Smoking	0.857 (0.463–1.588)	0.624			0.856 (0.514–1.427)	0.552		
ECOG	1.493 (0.720–3.097)	0.282			1.419 (0.771–2.613)	0.261		
PD-L1	0.501 (0.327–0.767)	0.001	0.599 (0.379–0.948)	0.029	0.733 (0.517–1.041)	0.083		
Pathology	0.896 (0.521–1.542)	0.693			1.036 (0.684–1.570)	0.866		
Maximum diameter	1.100 (1.020–1.186)	0.013	0.994 (0.900–1.098)	0.670	1.070 (0.998–1.147)	0.055		
T_ staging	1.161 (0.815–1.653)	0.409			1.028 (0.791–1.336)	0.836		
N_ staging	1.473 (0.929–2.336)	0.100			1.149 (0.819–1.610)	0.422		
*Number of metastases*								
0 versus 1–2	1.293 (0.539–3.104)	0.565			1.443 (0.728–2.861)	0.294		
0 versus > 2	1.466 (0.893–2.407)	0.130			1.541 (1.007–2.358)	0.047	1.229 (0.770–1.961)	0.217
1–2 versus > 2	1.614 (0.830–3.138)	0.158			1.832 (0.995–3.371)	0.052		
LDH levels	1.005 (1.002–1.007)	< 0.001	1.003 (0.999–1.006)	0.215	1.002 (1.000–1.004)	0.029	1.001 (0.998–1.003)	0.159
ALP levels	1.004 (1.002–1.006)	< 0.001	0.997 (0.994–1.001)	0.600	1.003 (1.000–1.005)	0.062		
β2-microglobulin levels	1.140 (0.790–1.646)	0.484			1.382 (1.016–1.880)	0.039	1.167 (0.805–1.693)	0.321
CEA levels	1.002 (1.000–1.003)	0.057			1.001 (0.999–1.003)	0.280		
CYRA 21-1 levels	1.005 (1.001–1.010)	0.014	1.003 (0.997–1.009)	0.352	1.005 (1.002–1.009)	0.006	1.006 (1.002–1.010)	0.005
SCC levels	0.993 (0.939–1.050)	0.810			1.031 (0.988–1.075)	0.155		
LDL-C levels	0.796 (0.569–1.113)	0.182			0.869 (0.655–1.153)	0.329		
HDL-C levels	1.059 (0.406–2.765)	0.906			0.963 (0.468–1.984)	0.919		
FFA levels	1.000 (0.999–1.001)	0.906			1.000 (0.999–1.001)	0.636		
PLR	1.002 (1.000–1.004)	0.021	1.001 (0.998–1.004)	0.176	1.001 (0.999–1.003)	0.422		
NLR	1.119 (1.038–1.205)	0.003	1.032 (0.915–1.165)	0.309	1.042 (0.977–1.112)	0.208		
HCT	0.945 (0.913–0.979)	0.002	0.934 (0.896–0.974)	0.001	0.963 (0.928–0.999)	0.044	0.967 (0.926–1.009)	0.221
Albumin levels	0.920 (0.870–0.973)	0.003	1.101 (0.919–1.320)	0.692	0.952 (0.913–0.992)	0.020	0.952 (0.913–0.991)	0.018
Fibrinogen quantification	1.004 (1.001–1.006)	< 0.001	0.996 (0.983–1.009)	0.986	1.001 (0.999–1.003)	0.208		
FARI	1.144 (1.072–1.220)	< 0.001	1.077 (1.003–1.157)	0.041	1.056 (0.998–1.116)	0.058		
NTRS	2.090 (1.531–2.853)	< 0.001	2.260 (1.559–3.276)	< 0.001	1.472 (1.126–1.924)	0.005	1.230 (0.824–1.837)	0.176

PD-L1, programmed cell death ligand 1; LDH, lactic dehydrogenase; ALP, alkaline phosphatase; CEA, carcinoma embryonic antigen; CYFRA21-1, cytokeratin 19 fragment; SCC, squamous cell carcinoma antigen; LDL-C, low-density lipoprotein cholesterol; HDL-C, high-density lipoprotein cholesterol, FFA, free fatty acid; PLR, platelet-lymphocyte ratio; NLR, neutrophil–lymphocyte ratio; HCT, hematocrit; FARI, fibrinogen–albumin ratio Index; NTRS, non-tumor-related scores

### Comparison of the NTRS in prognosis ability

**Fig. 1:**
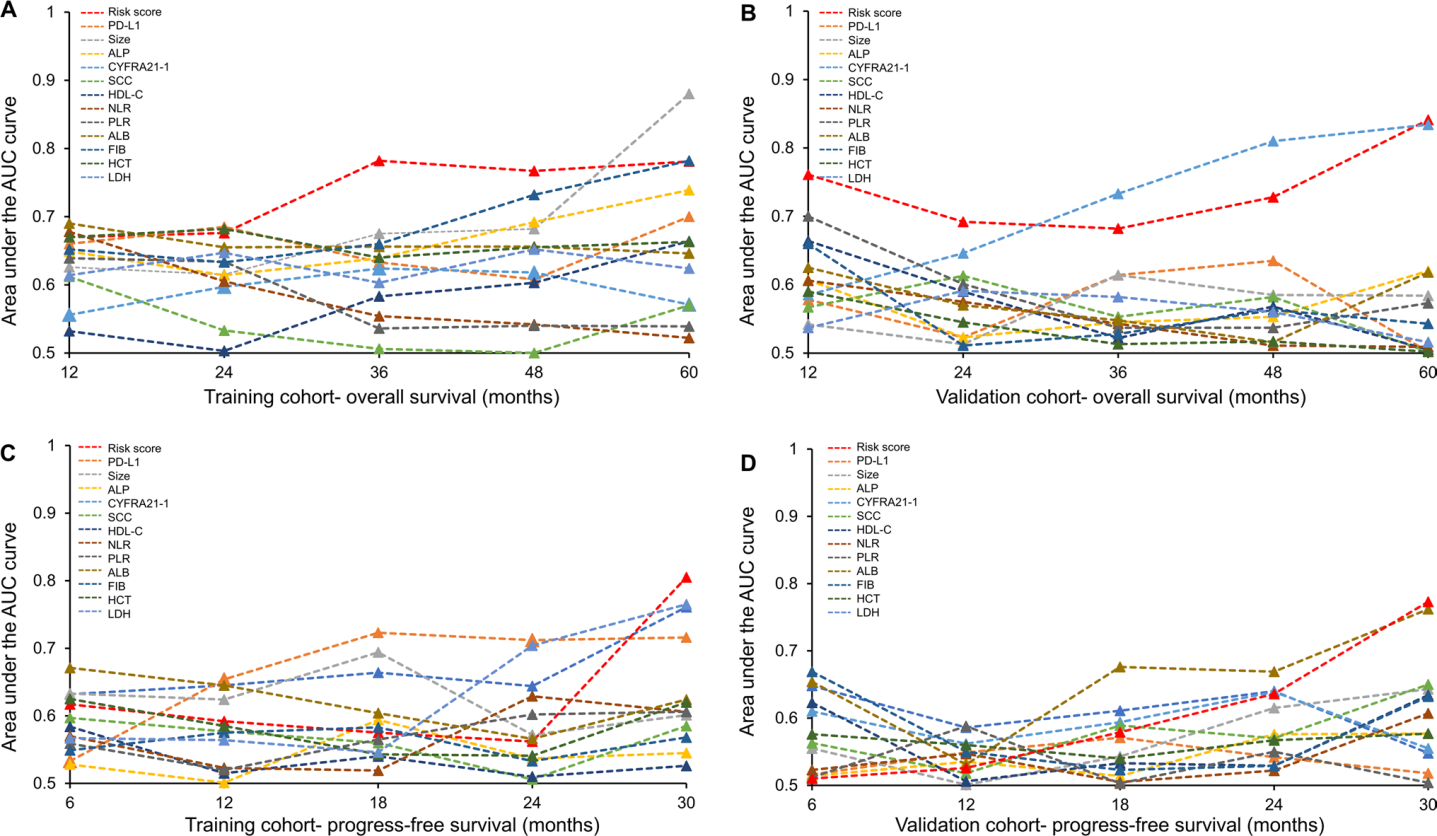
Time-dependent ROC curve analysis to compare the effect of NTRS, PD-L1 expression, tumor size, ALP, CYFRA21-1, SCC, HDL-C, NLR, PLR, ALB, FIB, HCT, and LDH in predicting **A**, **C** overall survival and **B**, **D** progression-free survival in **A**, **B** training cohort and **C**, **D** validation cohort. The vertical axis shows the corresponding area under the ROC curve at different time points, and the horizontal axis represents the time (months). PD-L1 programmed death ligand 1, ALP alkaline phosphatase, SCC squamous epithelial cell carcinoma-associated antigens, HDL-C high-density lipoprotein cholesterol, NLR neutrophil–lymphocyte ratio, PLR platelet-lymphocyte ratio, ALB albumin, FIB fibrinogen, HCT hematocrit, LDH lactic dehydrogenase, NTRS non-tumor-related scores

In the training cohort, time-dependent ROC analysis showed that the prognostic value of NTRS for OS was significantly better than PD-L1 expression (Wilcoxon matched pairs *P* = 0.012, 0.004) (Fig. 1A, B). The AUC of NTRS was significantly greater than PD-L1 expression at the 1, 2, 3, 4, 5-year time point. For example, in the training cohort (Fig. 1A), the C-index for 4-year OS probability prediction in NTRS was 0.767 (95% CI 0.636–0.859), which was significantly higher than PD-L1 expression (C-index 0.608, 95% CI 0.477–0.728); in the validation cohort (Fig. 1B), the C-index for 4-year OS in NTRS was 0.728 (95% CI 0.589–0.841), which was also greater than PD-L1 expression (C-index 0.635, 95% CI 0.492–0.763). However, the NTRS did not show better predictive value for PFS than PD-L1 expression in the training (*P* = 0.755) and validation groups (*P* = 0.503). In the training cohort, the 2-year PFS C-index was 0.644 (95% CI 0.546–0.768) for NTRS and 0.712 (95% CI 0.597–0.810) for PD-L1 expression; in the validation cohort the NTRS was 0.640 (95% CI 0.521–0.747) and PD-L1 expression was 0.542 (95% CI 0.424–0.657) (Fig. 1C, D). These results suggested that the NTRS was a better predictor of OS than PD-L1 expression.

### OS and PFS assessed by the NTRS and PD-L1 expression

**Fig. 2:**
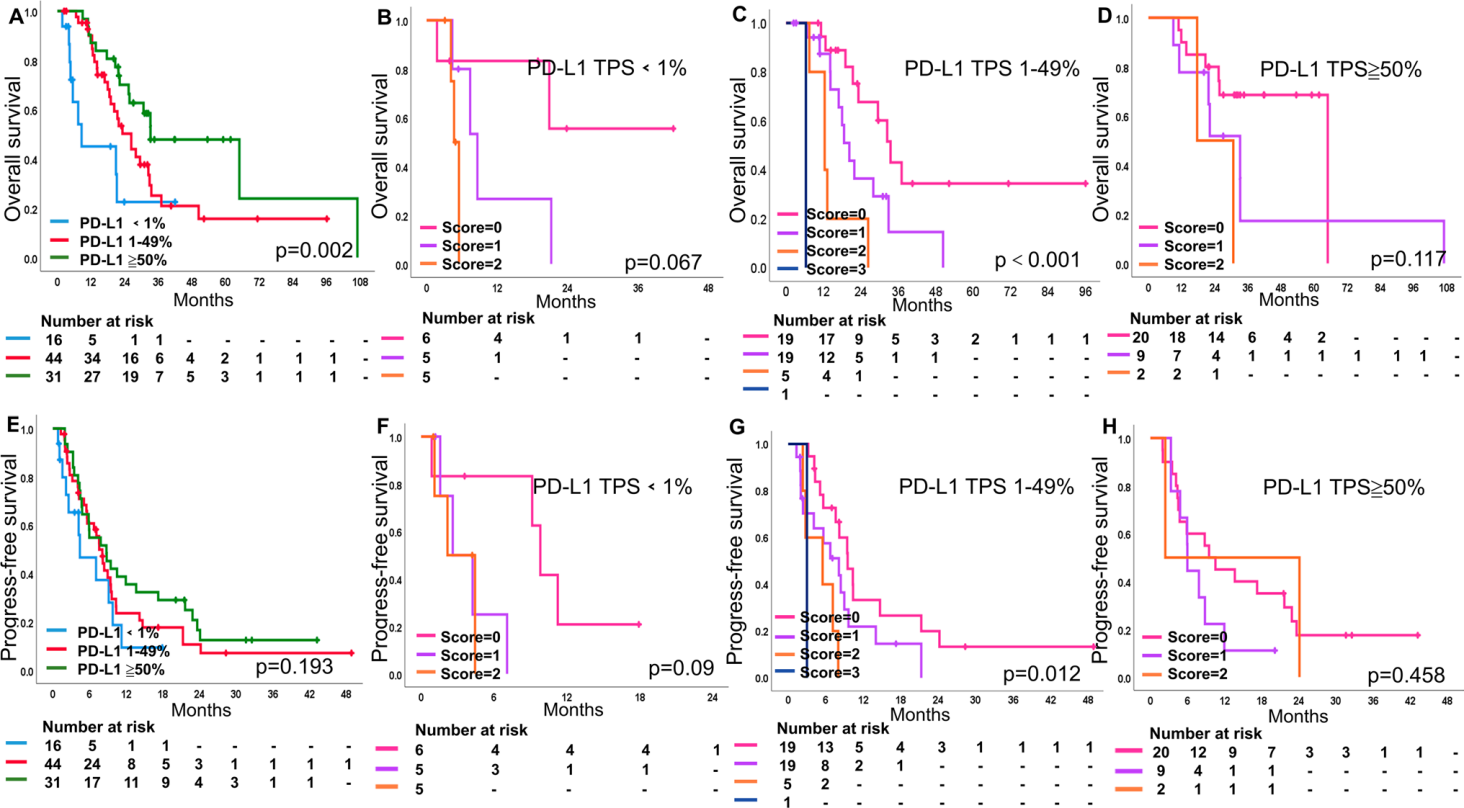
**A**–**D** OS and **E**–**H** PFS in **A**, **E** PD-L1 (< 1%, 1–49%, ≧ 50%) expression and different risk score (score = 0, 1, 2, 3) in **B**, **F** PD-L1 < 1% group, **C**, **G** PD-L1 1–49% group and **D**, **H** PD-L1 ≧ 50% group in training cohort. The number and *P*-value can be seen clearly. A *P*-value of ≦ 0.05 was considered significant. PD-L1 programmed death ligand 1, OS overall survival, PFS progress-free survival

Figures 2 and S3 showed OS and PFS for different scores (0–3) of PD-L1 expression and NTRS stratification in the training and validation cohorts were shown. Survival curves for NTRS were more discriminatory compared to PD-L1 expression.

In the training cohort, mOS was 8.6, 26.3 and 33.2 months for patients with PD-L1 expression < 1%, 1–49% and ≧ 50%, respectively. mOS was 64.9, 21.1, 12.4 and 6.3 months for patients with NTRS of 0–3, respectively (Figs. 2A, S3A). In patients with PD-L1 1–49%, mOS was statistically different at 33.5, 20.3, 12.4 and 6.3 months when score = 0, 1, 2, and 3, respectively (*P* < 0.001) (Fig. 2C), but no survival differences was seen among scores of PD-L1 < 1% and ≧ 50% (Fig. 2B, D). mPFS for PD-L1 expression was 4.4, 7.6 and 8.8 months and mPFS for NTRS were 9.8, 5.9, 4.4 and 3.2 months, respectively (Figs. 2E, S3B). Differences in PFS were seen in patients with PD-L1 1–49% when scores = 0, 1, 2, and 3 (*P* = 0.012), while no differences were seen in patients with PD-L1 < 1% and ≧ 50% (Fig. 2F–H).

**Fig. 3:**
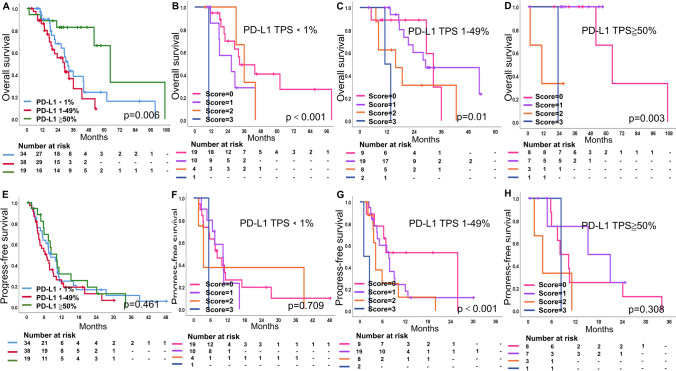
**A**–**D** OS and **E**–**H** PFS in **A**, **E** PD-L1 (< 1%, 1–49%, ≧ 50%) expression and different risk score (score = 0, 1, 2, 3) in **B**, **F** PD-L1 < 1% group, **C**, **G** PD-L1 1–49% group and **D**, **H** PD-L1 ≧ 50% group in validation cohort. The number and *P*-value can be seen clearly. A *P*-value of ≦ 0.05 was considered significant. PD-L1 programmed death ligand 1, OS overall survival, PFS progress-free survival

In the validation cohort, mOS was 31.7, 29.1 and 64.6 months for patients with PD-L1 expression < 1%, 1–49% and ≧ 50%, respectively, and 43.6, 44.0, 18.7 and 11.1 months for patients with scores 0–3 (Figs. 3A, S3C). In the stratification of PD-L1, OS was difference when risk score = 0, 1, 2, 3 (*P* < 0.001, 0.01, and 0.003, respectively) (Fig. 3B–D). mPFS for PD-L1 expression was 8.3, 6.9 and 10.3 months; while, mPFS for NTRS was 8.3, 7.9, 4.2, and 2.5 months, respectively (Figs. 3E, S3D). When risk score = 0, 1, 2, 3, there was a difference in PFS in patients with PD-L1 1–49%, while no difference was seen in patients with PD-L1 < 1% and ≧ 50% (Fig. 3F–H).

In pooled cohort, OS was statistically different when PD-L1 expression was stratified at < 1%, 1–49% and ≧ 50% (mOS by score = 0, 1, 2, 3. mOS at PD-L1 < 1%: 32.5 m, 18.0 m, 30.1 m, 10.2 m, *P* = 0.013; mOS at PD-L1 1–49%: 32.5 m, 23.5 m, 13.2 m, 13.6 m, *P* = 0.001; mOS at PD-L1≧ 50%: 64.6 m, 33.2 m, 17.6 m, 23.1 m, *P* = 0.006). In summary, among patients with PD-L1 1–49%, the higher the score, the worse the PFS and there was a statistical difference (*P* = 0.407 for PD-L1 < 1%; *P* = 0.011 for PD-L1 1–49%; *P* = 0.618 for PD-L1≧ 50%) (Fig. S4).

Thus, even though PD-L1 had statistically different OS in the < 1%, 1–49%, and ≧ 50% groups, there was no PFS difference. Moreover, both OS and PFS were statistically different in the PD-L1 1–49% stratification in each NRTS score (0–3) in both the training and validation cohorts.

### NTRS showed higher efficiency than PD-L1 in predicting response

**Fig. 4:**
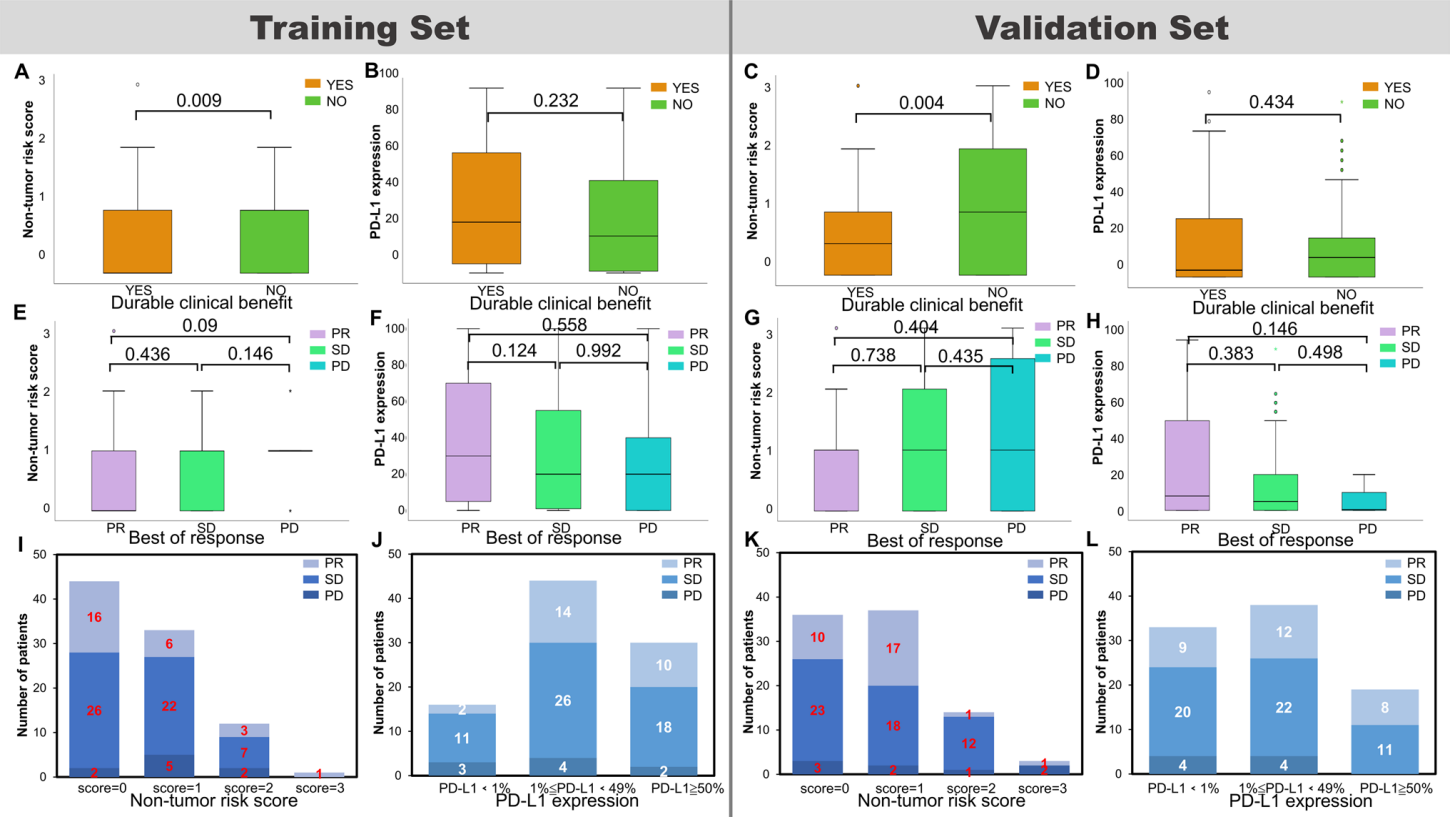
Comparisons between PD-L1 expression and NTRS e in predicting response to ICIs in NSCLC in **A**, **B**, **E**, **F**, **I**, **J** training cohort and **C**, **D**, **G**, **H**, **K**, **L** validation cohort. Differential analyses of **A**, **C** NTRS and **B**, **D** PD-L1 expression among patients with durable clinical benefit. Differential analyses of **E**, **G** NTRS and **F**, **H** PD-L1 expression among PR/SD/PD patients. Number of patients with PR/SD/PD responses among **I**, **K** PD-L1 expression and **J**, **L** NTRS in **I**, **J** training cohort and **K**, **L** validation cohort. PD-L1 programmed death ligand 1, NTRS non-tumor-related scores, ICIs immune checkpoint inhibitors, NSCLC non-small cell lung cancer, PR partial response, SD stable disease, PD progressed disease

NTRS demonstrated a higher efficiency than PD-L1 expression as a biomarker for immunotherapy. When response was evaluated with RECIST1.1, NTRS were more different between the DCB and NDB groups than PD-L1 expression (Fig. 4A–D), but there was no significant difference between the BOR and non-BOR groups (Fig. 4E–H). The proportion of NTRS and PD-L1 was 90% (*N* = 81) and 90% (*N* = 81) for non-PD (PR + SD) patients in the training cohort and 92% (*N* = 83) and 91% (*N* = 82) for non-PD patients in the validation cohort (Fig. 4I–L).

ROC analysis demonstrated that NTRS significantly enhanced the predictive efficiency of PD-L1 expression (Fig. S5). When BOR was assessed in the training cohort, the AUC of NTRS was 0.650, which was the trend of assessing prognosis than 0.522 of PD-L1 (Figs. S5A, 5E). In evaluation of DCB in the training cohort, the AUC of NTRS was higher, 0.644, than 0.572 for PD-L1 (Fig. S5B, 5E). When BOR was assessed in the validation cohort, the AUC for NTRS was 0.584, significantly lower than 0.637 for PD-L1 (Figs. S5C, 5E). In evaluation of DCB in the validation cohort, the AUC of NTRS was highest at 0.665, which was higher than that of 0.547 for PD-L1 (Fig. S5D, 5E). Taken together, the combination of PD-L1 expression and NTRS had a greater AUC and higher predictive prognosis value to improve the effectiveness of PD-L1 expression as an immunotherapy biomarker.

### Distribution and classification characteristics of NTRS and PD-L1 expression in metastatic sites and tumorigenic factors

The distribution of PD-L1 expression in tissue samples from different metastatic sites was moderately different depending on organ differences (Fig. S6). Among metastatic sites, the proportion of patients with high PD-L1 expression was greatest in lung (PD-L1 (≧ 50%) = 29%) and smallest in pleural (PD-L1 (≧ 50%) = 13%), and the proportion of patients was greatest in the adrenals (scores (1 + 2 + 3) of 66%) and smallest in the lung (scores (1 + 2 + 3) of 29%) (Fig. S6A, B). Differences in the distribution of PD-L1 expression and NTRS at different metastatic sites were further explored. PD-L1 status (PD-L1≧ 1% vs. < 1%) was predictive of ICI treatment response in brain metastases, but no significance was found between metastasis site and treatment response in the risk score (Fig. S6C, D).

**Fig. 5:**
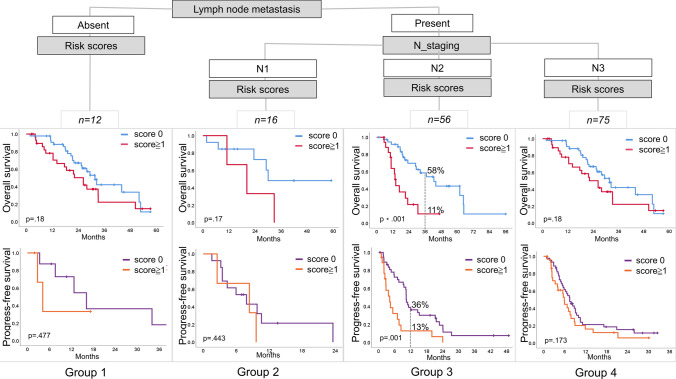
A Kaplan–Meier curve model comparing the hierarchical association of prediction factors associated with OS and PFS among patients with LNMs. OS overall survival, PFS progress-free survival, LNM lymph node metastases

Categorical and regression analyses were used to assess the prognostic factors of NTRS and PD-L1 expression in long-term survival. Table S2 provided the tumor correlates of NTRS and PD-L1 expression. In PD-L1 expression, the level of the tumor marker SCC was identified as the most important correlate, while in the NTRS, LNMs status was determined as the most important correlate. And the critical ranges of LNMs and SCC levels were identified as N2 and 2.5 ng/ml. Specifically, regression analysis of critical values in the PD-L1≧ 50% group suggested worse PFS when SCC levels exceeded 2.5 ng/ml (mPFS: 8.4 vs. 2.4 months, *P* < 0.001), but no difference was found in OS (Fig. S7). In addition, in the NTRS, LNMs suggested survival differences in OS and PFS (Fig. 5). In particular, N2 staging resulted in shorter OS and PFS in this group of patients with NTRS ≧ 1 (mOS: 42.6 vs. 14.2 months, *P* < 0.001; mPFS: 10.37 vs. 4.17 months, *P* = 0.001). These results implied longer OS in patients with PD-L1 ≥ 1% brain metastases, but there was no correlation between NRTS and metastatic site. In a further, patients with NTRS (≧ 1, host factor) and LNMs (N2, tumorigenic factor) had worse OS and PFS; patients with PD-L1 (≧ 50%) and SCC levels (> 2.5 ng/mL) had shorter PFS.

### Subgroup analyses of OS and PFS based on NTRS and PD-L1 expression

**Fig. 6:**
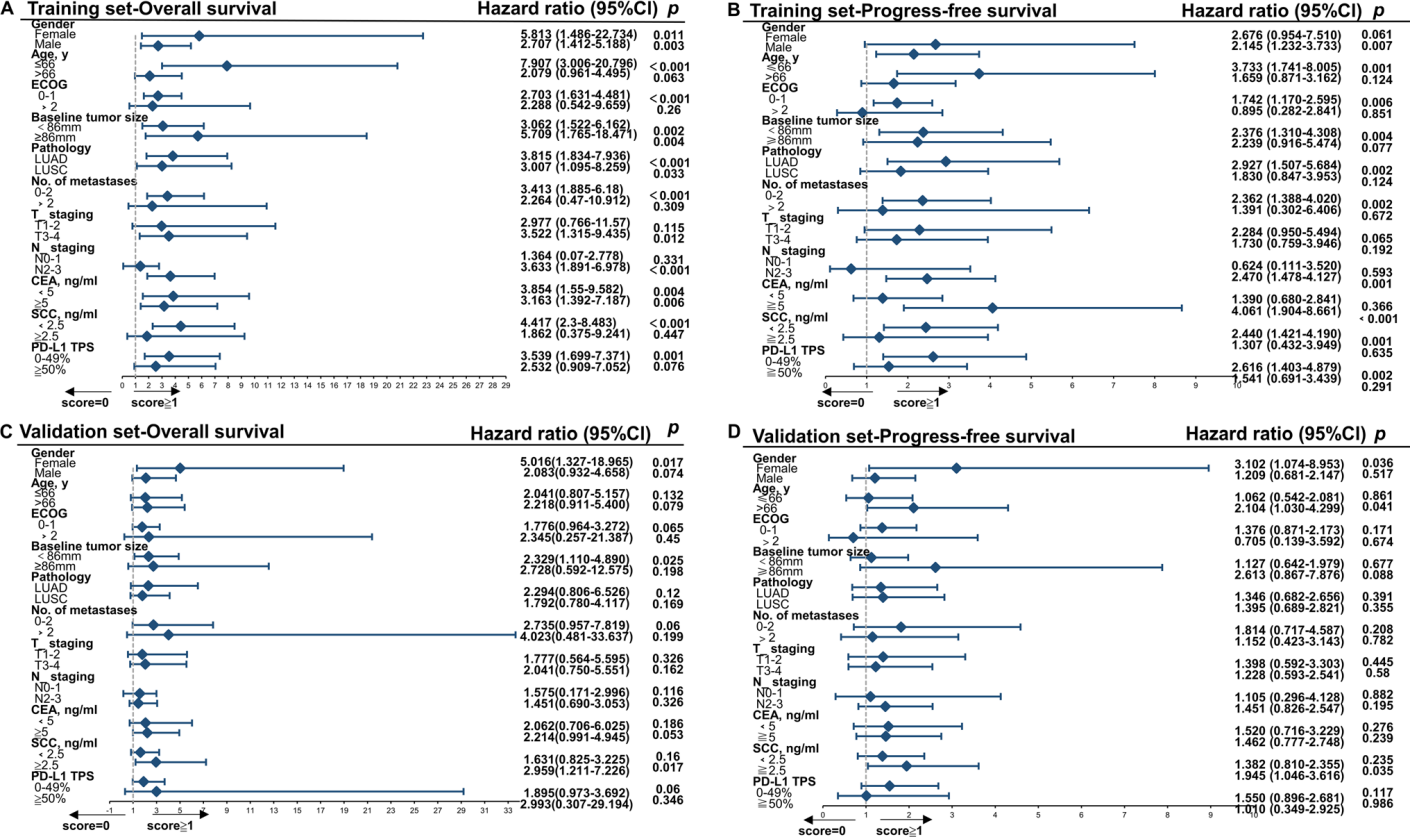
Subgroup analysis using univariate Cox regression was performed to assess the ability of NTRS to discriminate **A**, **C** OS and **B**, **D** PFS in patients with different clinical characteristics in **A**, **B** training cohort and **C**, **D** validation cohort. OS overall survival, PFS progress-free survival, CI confidence intervals, ECOG Eastern Oncology Collaborative Group, TKI tyrosine kinase inhibitors, LUAD Lung adenocarcinoma, LUSC Lung squamous cell carcinoma, CEA carcinoma embryonic antigen, SCC squamous cell carcinoma antigen, PD-1 programmed cell death protein 1, TPS tumor proportion score, NTRS non-tumor-related scores

Patients with NTRS scores (0 vs. ≧ 1) (Fig. 6) and PD-L1 expression (0 vs. ≧ 1) (Fig. S8) were examined for differences in OS and PFS in the training and validation cohorts, respectively. When risk scores ≧ 1, OS was significantly shorter for both risk factors (female: *P* = 0.01; size < 86 mm: *P* < 0.025) (Fig. 6A, C), but PFS was not uniform for these factors in the training and validation cohorts (Fig. 6B, D). Furthermore, in the five factors of male, size < 86 mm, ECOG 0–1, N2-3, and SCC level less than 2.5, OS was statistically prolonged despite the absence of positive expression of PD-L1 in the training and validation cohorts (Fig. S8). To summarize, OS shortened with increasing PD-L1 levels and NTRS, but no similar results for PFS. Also, there are 5 factors (male, size < 86 mm, ECOG 0-1, N2-3, SCC level < 2.5) that indicated an extended OS.

## Discussion

ICIs have been fundamentally altering the administration of NSCLC. Despite the success of ICIs in a proportion of NSCLC patients, some patients continue to progress and die in a relatively short-term period. A major challenge is to establish which patients will respond beneficially to ICIs and have a longer prognosis. This paper has provided an alternative foundation for the analysis of the prognostic and predictive value of NTRS that could improve more significant survival and benefit from ICIs in a fuzzy environment.

Accordingly, most biomarkers or risk model recommendations were extrapolated from subgroup analysis of clinical trials or some institutions' experiences with solid tumors, including NSCLC [14–17, 21, 24–36]. ECOG ≦ 2 [21, 33], men (single-agent ICI) [29, 30], women (combination of ICI and chemotherapy) [31], BTS [24], smoking status [25] and BMI ≧ 25 [32] suggest a longer prognosis. Malignant pleural effusion [27, 33], brain and liver metastases [27, 28], NLR [34, 35], high levels of LDH [36], c-reactive protein (CRP) [33], and fibrinogen–albumin ratio index (FARI) [26] were independent poor prognostic factors. However, it was poorly difficult to identify patients who benefit from ICIs by biomarkers alone due to confounding factors.

Emerging studies of risk models [14–19] have been introduced to predict the risk of NSCLC progression. The survival neural network model investigated more promising results in the prediction of lung cancer-specific survival than the tumor, node, and metastasis stage (C statistic = 0.739 vs. 0.706) [16]. According to a model based on ferroptosis-related genes, a series of bioinformatics analyses were conducted to verify the predictive ability of OS (*P* < 0.001 vs. 0.0095, AUC = 0.679 vs. 0.618 in ≧ high and low groups between training and validation cohort) as independent prognostic factors (HR 0.63, 95% CI 0.44–0.91, *P* = 0.01) for NSCLC patients with immunosuppressive status [14]. High tumor microenvironment related genes model was identified to predict longer OS (log-rank *P* = 0.044) and was an independent factor (HR 3.32, 95% CI 2.16–5.09) [15]. CT-based imaging signs, systemic inflammatory response index, prognostic nutritional index and CEA were filtered and included in the nomogram (AUC 0.828, 95% CI 0.778–0.877 in the training cohort, AUC 0.816, 95% CI 0.737–0.895 in the validation cohort, respectively) for predicting LNMs in NSCLC patients [17]. Patients were classified into good (0–8) and poor (≧ 9) groups based on clinical prognostic model (3 (if male) + 3 (if ECOG = 1) + 8 (if ECOG = 2) + 11 (if initial stage = IV) + 4 (if weight loss)) [19]. The median OS in the two groups were 13.15 (95% CI 10.82–15.91) and 8.52 months (95% CI 7.5–9.63), respectively [19]. A high HALP score (hemoglobin (g/L) × albumin (g/L) × lymphocytes (g/L)/platelets (g/L)) were significantly associated with lower OS (HR 0.707, 95% CI 0.503–0.995) and DFS (HR 0.671, 95% CI 0.491–0.916) [18]. Unfortunately, these models are either based on bioinformatics analysis, which inevitably increases patient costs; Or, age-specific prognostic models fail to fully account for serologically specific factors and PD-L1 expression. Contrasted and these models, the advantages of NTRS are high advantage/cost and simple admittance to explicit markers in the determination factors. These assist clinicians with making a fast evaluation in light of the serological indicators of the patient.

Apart from the above, the data in these studies [14–17, 21, 24–36] have only highlighted the value of tumor or non-tumor-related biomarkers or models as single or independent factors for OS, PFS, therapeutic approaches and clinical responses in NSCLC patients, but have ignored the improvement in prognosis and prediction when combined with PD-L1 expression. Our study suggested that the risk score could more accurately predict PFS and OS in combination with PD-L1 expression in both the training and validation cohorts. This result has rapidly and easily complemented the application and reference significance of PD-L1 expression for clinical practice and has improved the predictive accuracy of OS and PFS. At present, PD-L1 is the main biomarker to judge the use of immunotherapy. Contrasted the above prognostic models, the combination of NTRS and PD-L1 expression can assess prognosis and response, instead of depending on a prognostic model alone. This is the main significance of the establishment of NTRS.

Importantly, several studies [37, 38] demonstrated that predicting DCB was preferable in a number of scores/models that were built on strong economic conditions and methodologies. Among them, three genomic biomarkers, namely TMB, intratumoral heterogeneity and loss of heterozygosity in human leukocyte antigen, and the support vector machine (SVM)_poly method were used to construct a DCB prediction model. The DCB multi-feature model exhibited better predictive value with AUC = 0.77 (cohort1) and 0.78 (cohort2) [37]. Another study [38] illustrated that a novel 18 mutated genes score 18 (TMS18) (HR 0.307, *P* < 0.001) had smaller HR and P value than PD-L1 expression (HR 0.403, *P* = 0.005) in survival analysis in 240 NSCLC patients. Moreover, the AUC of TMS18 was significantly higher than that of TMB, and the combination of TMS18 and PD-L1 improved the accuracy. The above studies were performed on the basis of TMB-related markers or genes, which made it more difficult to put into clinical practice. Our analysis suggested that the risk scores are more valuable than PD-L1 expression alone in predicting DCB. The model scores provide simple and direct predictive factors and ranges of values that can effectively distinguish DCB.

Furthermore, primary tumor LNM status [17], number and size of tumors [24], PD-L1 expression status [10, 11], and ECOG [21, 33, 39] have been identified as important discriminatory variables in patients treated with ICIs. These studies have displayed the prognostic and predictive value of these factors, but few papers have examined the relative importance of "celebrity" markers in combination with other factors for survival. In the era of ICIs, the optimal cutoff value for assessing a prognostic factor may have changed. One possible explanation is related to the different disease burden of patients. A classification and regression tree model identified presence of LNMs (single or 2 vs. ≧ 3) as the most important prognostic factor among wild-KRAS patients; whereas, CEA levels (< 6 ng/mL, ≧ 6 ng/mL and < 360 ng/mL, and ≧ 360 ng/mL) were identified as the most important prognostic factor in mutation-KRAS patients [40]. This study demonstrated that two separate factors or two levels of a biomarker can be used as the optimal cutoff value for prognostic assessment. However, these have been less studied in NSCLC. Of note, in patients stratified by risk score, primary tumor LNM constituted the only prognostic “node” for OS (Fig. 5); and SCC levels (“ < 2.5 ng/mL” and “≧ 2.5 ng/mL”) were the most important prognostic risk factors in patients stratified by PD-L1 expression (Fig. S7). The possible reason was that patients with N2 metastases have more extensive metastatic events; whereas, patients with N3 metastases were highlighted by LNMs and had relatively less distant tumor burdens. In contrast, patients with SCC ≧ 2.5 ng/mL and PD-L1 ≧ 50% had less PFS benefit. The result may be explained that these patients had more tumorigenic factors leading to worse prognosis, diluting the prognosis based on PD-L1 expression alone.

Interestingly, previous studies have observed that malignant pleural effusion [27] (HR 1.52, 95% CI 1.01–2.29, *P* = 0.043) were independent predictors of shorter PFS even though patients’ PD-L1 TPS was high. Additionally, PD-L1 expression was highest in adrenals, livers and LNMs, and lower in bone and brain metastases [28]. In our study, we observed higher PD-L1 expression in the LNMs and lower PD-L1 expression in the pleural effusions and adrenals, compared with higher risk scores in adrenals, livers and LNMs and lower in bones. These responses might be influenced by small sample sizes, multifocal metastases, and inconsistent metastatic burdens. Another study [41] demonstrated a 30% response rate to a single ICI in brain metastases in patients with driver-negative, high PD-L1 expression and low CNS disease burden. Our institution [26] has previously proved that high FARI was associated with poorer OS in patients with bone metastases (*P* = 0.03). Patients with brain metastases with PD-L1 expression > 1% had a longer OS, which was consistent with previous studies, but there was no prognostic difference between the score level and frequent metastatic sites. This may be due to the absence of correlation between the new risk score and metastatic sites, or the inability to effectively assess the metastatic characteristics of patients. The development of biomarkers or risk models remains an active area of investigation.

The three indexes in NTRS are ALP, CYFRA21-1 and HDL-C. Many studies have shown that elevated ALP is a robust adverse prognostic factor for survival in metastatic neuroendocrine tumors [42], spinal metastatic diseases [43], hepatic metastases [44], etc. These metastases predicted worse survival times. CYFRA21-1 has been associated with metastasis and poor prognosis [45]. CYFRA21-1 levels had stronger association with metastases and survival status than CEA and NSE in all lung cancer patients [46]. In this research, Cox regression analyses showed that only CYFRA21-1 was an independent prognostic factor in ADC and SCLC patient subtypes. Current literature suggests a positive association between cholesterol levels and the risk and progress of cancer development [47]. This is principally because of the way that raised HDL-C gives the energy metabolism and membranous components expected by malignant growth cells to advance cancer proliferation and improvement. Consequently, the rationale is poor indicators that promotes tumor proliferation and metastasis behind selecting the specific factors.

Limitations of this study include the retrospective design, single-institution experiences, small samples, and restrictions of the electronic case system. Also, patients with ECOG ≧ 2 were treated with a single ICIs, similar to that of patients with PD-L1 expression ≧ 50%, which had a confounding prognostic and predictive implications. Second, existing studies on this topic are lacking, although the value of FARI as a prognostic biomarker in the assessment of patients with solid tumors receiving immunotherapy was explored. Third, diets were standardized prior to laboratory testing, but patients with combined insidious metabolic syndrome could not be completely excluded. Last, the limited time available led to incomplete sections of the survival data.

There are several strengths in this study. It’s a new prognostic and predictive model and served as an independent poor prognostic factor for patients with advanced NSCLC undergoing ICIs based on PD-L1 expression or ECOG status as monotherapy or combination chemotherapy. And this model combined with PD-L1 expression could more accurately improve and complement the assessment of prognosis and prediction. Furthermore, when this model was combined with tumor-related factors, a score of ≧ 1 was associated with shorter OS and PFS for LNMs (N2). The essential meaning of determining the NTRS is the combination of PD-L1 expression to completely assess the prognosis and treatment responsiveness of NSCLC patients, as well as predominant benefit/cost proportion.

## Conclusions

In summary, a simpler, faster and more accessible NTRS was developed compared to PD-L1 as a prognostic and predictive model for advanced NSCLC patients treated with ICIs compared to PD-L1. The score consisting of 3 non-tumorigenic blood factors may provide a universal approach to improve biomarkers in NSCLC patients applying only PD-L1 expression alone and will hopefully serve as useful feedback information for improvements for prediction and prognosis of ICIs in advanced NSCLC. However, future prospective studies are required to confirm this conclusion due to the study's small samples.

## Supplementary Information

Below is the link to the electronic supplementary material.

Supplementary file1 (DOCX 1440 kb)

Supplementary file2 (DOCX 26 kb)

## Data Availability

The datasets generated and analyzed during the current study are available from the corresponding author on reasonable request.

## Abbreviations

NSCLC: Non-small cell lung cancer
PD-L1: Programmed death ligand 1
ICIs: Immune checkpoint inhibitors
ROC: Receiver operating characteristic
NTRS: Non-tumor-related scores
OS: Overall survival
PFS: Progress-free survival
DCB: Durable clinical benefit
TPS: Tumor percentage scores
TMB: Tumor mutational burden
MSI: Microsatellite instability
dMMR: Defective mismatch repair
ORR: Objective response rate
LNMs: Lymph node metastases
RECIST: Response evaluation criteria in solid tumors
ECOG: Eastern Cooperative Oncology Group
BTS: Baseline tumor size
BOR: Best overall response
HCT: Hematocrit
LDH: Lactate dehydrogenase
ULN: The upper limit of normal
ALP: Alkaline phosphatase
ALB: Albumin
LLN: The lower limit of normal
HDL-C: High-density lipoprotein cholesterol
LDL-C: Low-density lipoprotein cholesterol
FIB: Fibrinogen
CEA: Carcinoembryonic antigen
SCC: Squamous epithelial cell carcinoma-associated antigens
CT: Computed tomography
MRI: Magnetic resonance imaging
AUC: Area under the curve
NCCN: National Comprehensive Cancer Network
CR: Complete response
PR: Partial response
SD: Stable disease
NDB: No durable benefit
PD: Progressed disease
IQRs: Interquartile ranges
NLR: High neutrophil lymphocyte ratio
PLR: Platelets lymphocyte ratio
CI: Confidence interval
CRP: C-reactive protein
FARI: Fibrinogen–albumin ratio index
SVM: Support vector machine
TMS18: Mutated genes score 18

## Declarations

### Permission to reproduce material from other sources

Not available.

### Clinical trial registration

Not available.
