# Supplementary material for: Non-tumor-related prognostic factors for immunotherapy–chemotherapy or immunotherapy alone as first-line in advanced non-small cell lung cancer (NSCLC)
Source: Clin Exp Med. 2024 Mar 15;24(1):52. doi: 10.1007/s10238-024-01298-z (PMC10942875; doi:10.1007/s10238-024-01298-z)
Supplement: Supplementary file 1 — Supplementary file1 (DOCX 1440 kb) [file 10238_2024_1298_MOESM1_ESM.docx]

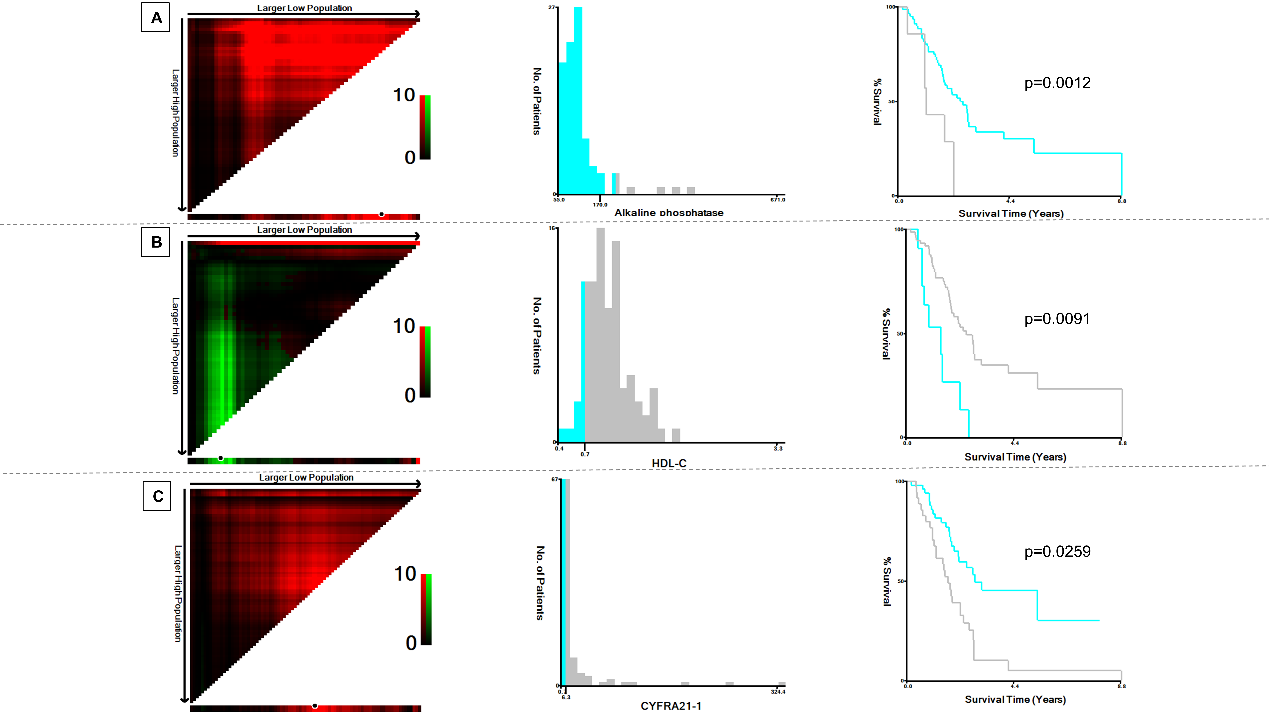


**Fig. S1**

X-title analysis to determine the optimal cut-off values of (**A**) ALP, (**B**) HDL-C, and (**C**) CYFRA21-1 in training cohort. ALP alkaline phosphatase, HDL-C high density lipoprotein cholesterol, CYFRA21-1 cytokeratin 19 fragment.


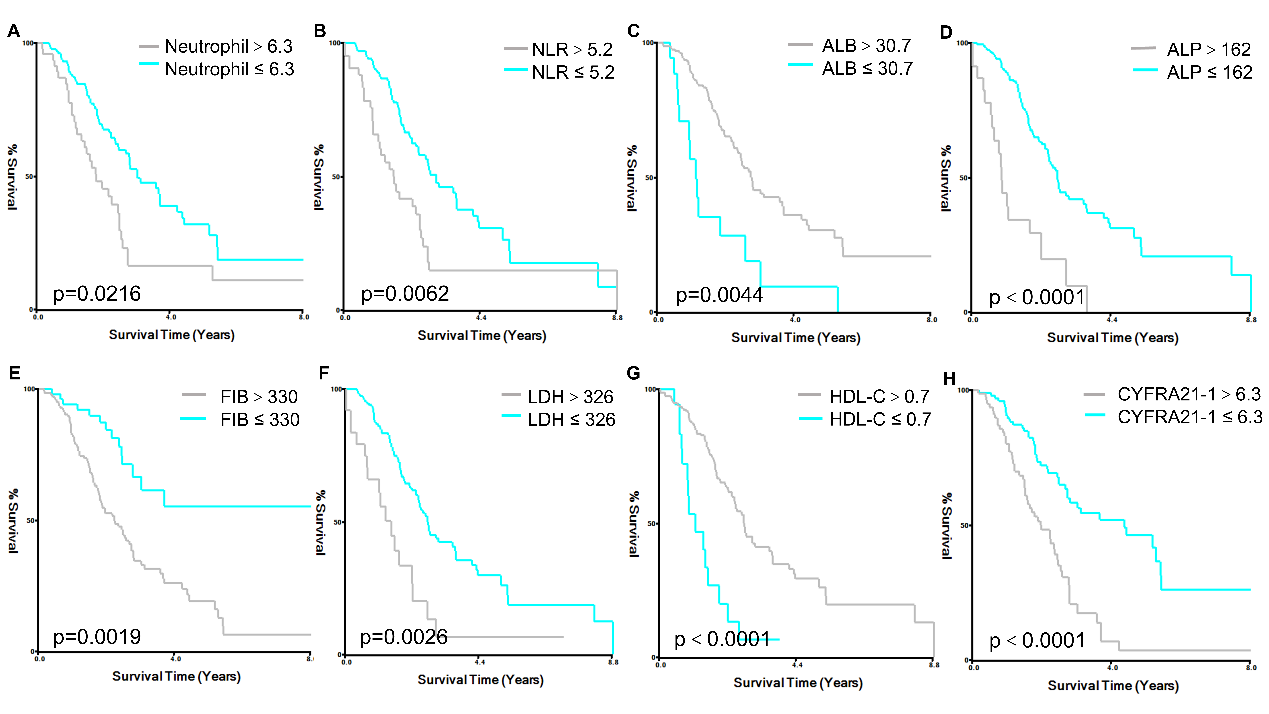


**Fig. S2**

X-title analysis in pooled cohort about (**A**) neutrophil, (**B**) NLR, (**C**) ALB, (**D**) ALP, (**E**) FIB, (**F**) LDH, (**G**) HDL-C, (**H**) CYFRA21-1. The significant OS about these eight markers was presented. NLR neutrophil-lymphocyte ratio, ALB albumin, ALP alkaline phosphatase, FIB fibrinogen, LDH lactate dehydrogenase, HDL-C high density lipoprotein cholesterol, CYFRA21-1 cytokeratin 19 fragment. OS overall survival.


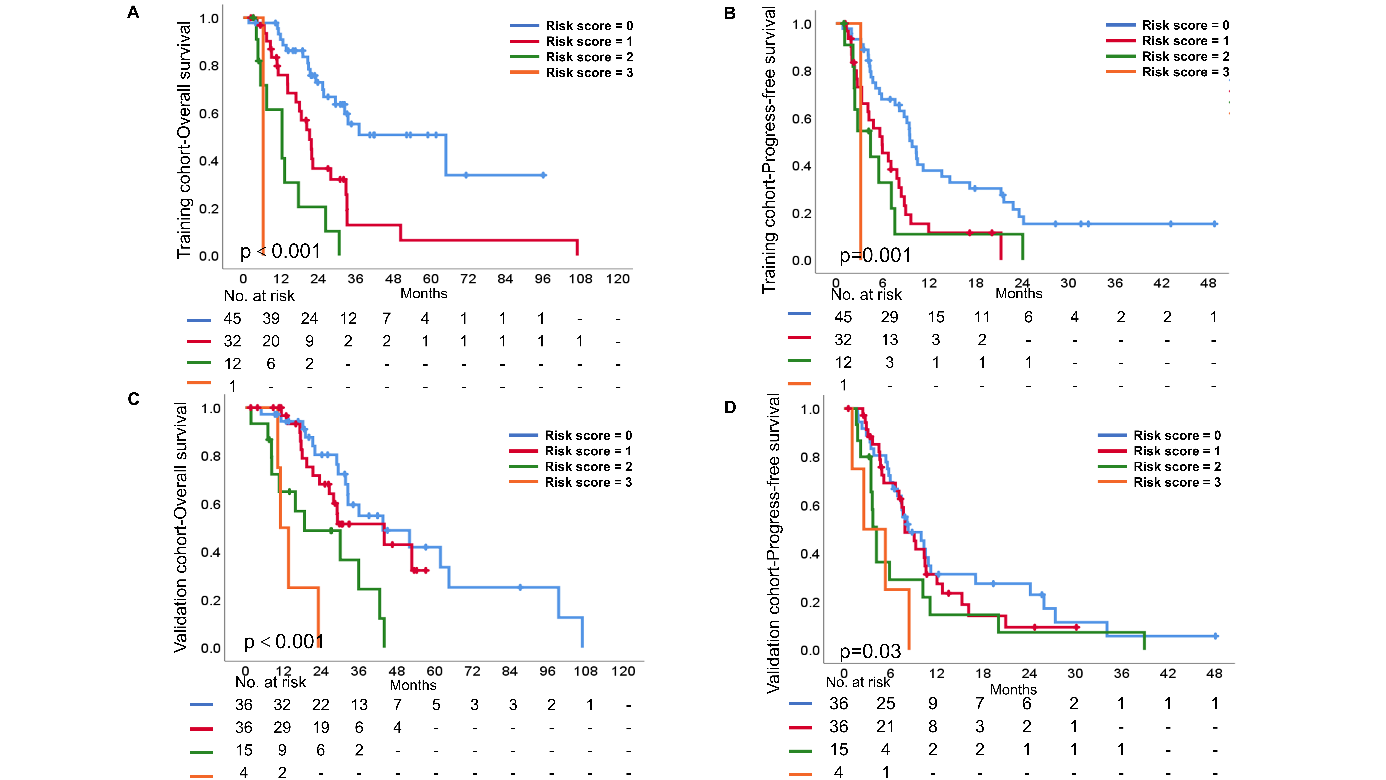


**Fig. S3**

(**A**, **C**) OS and (**B**, **D**) PFS in different risk score groups between (**A**, **B**) training cohort and (**C**, **D**) validation cohort. (**A**) OS in training cohort, (**B**) PFS in training cohort, (**C**) OS in validation cohort, and (**D**) PFS in validation cohort were present. The number and P-value can be seen clearly. A P-value of ≦0.05 was considered significant. OS overall survival, PFS progress-free survival.


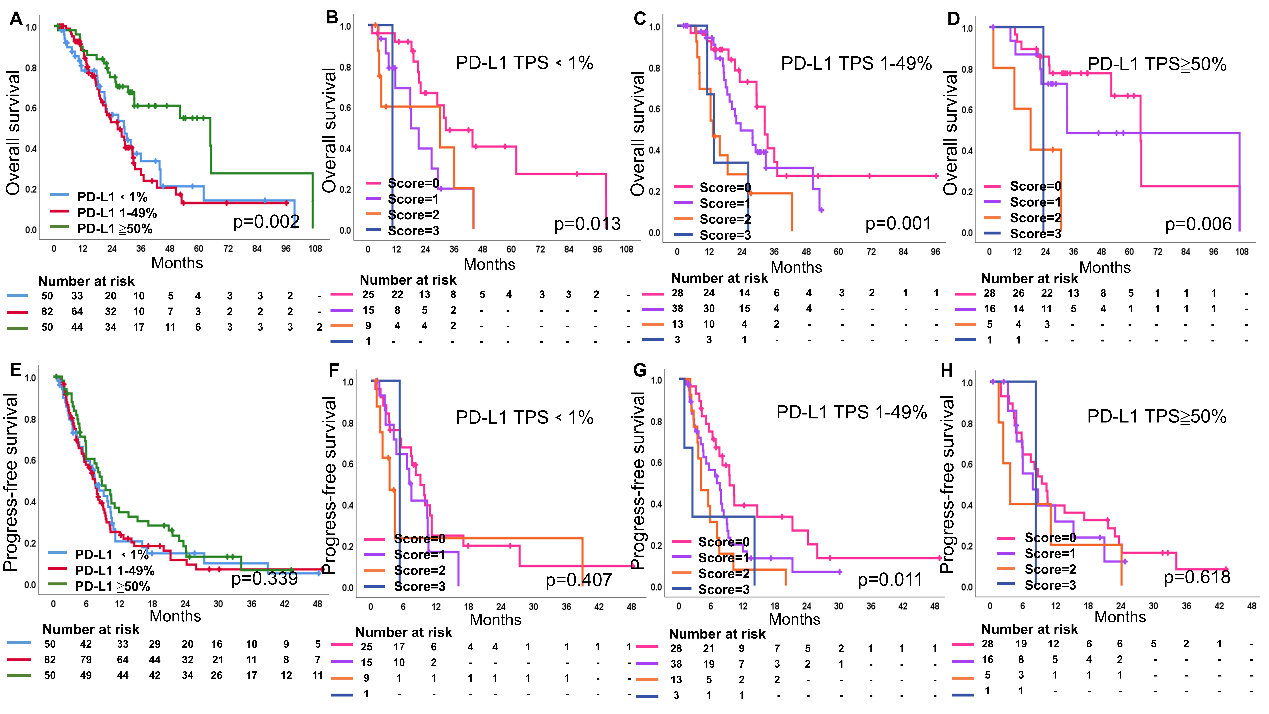


**Fig. S4**

(**A**, **B, C, D**) OS and (**E**, **F, G, H**) PFS in (**A**, **E**) PD-L1 (＜1%, 1-49%, ≧50%) expression and different risk score (score=0, 1, 2, 3) in (**B**, **F**) PD-L1 ＜1% group, (**C**, **G**) PD-L1 1-49% group and (**D**, **H**) PD-L1 ≧50% group in pooled patients. The number and P-value can be seen clearly. A P-value of ≦0.05 was considered significant. PD-L1 programmed death ligand 1, OS overall survival, PFS progress-free survival.


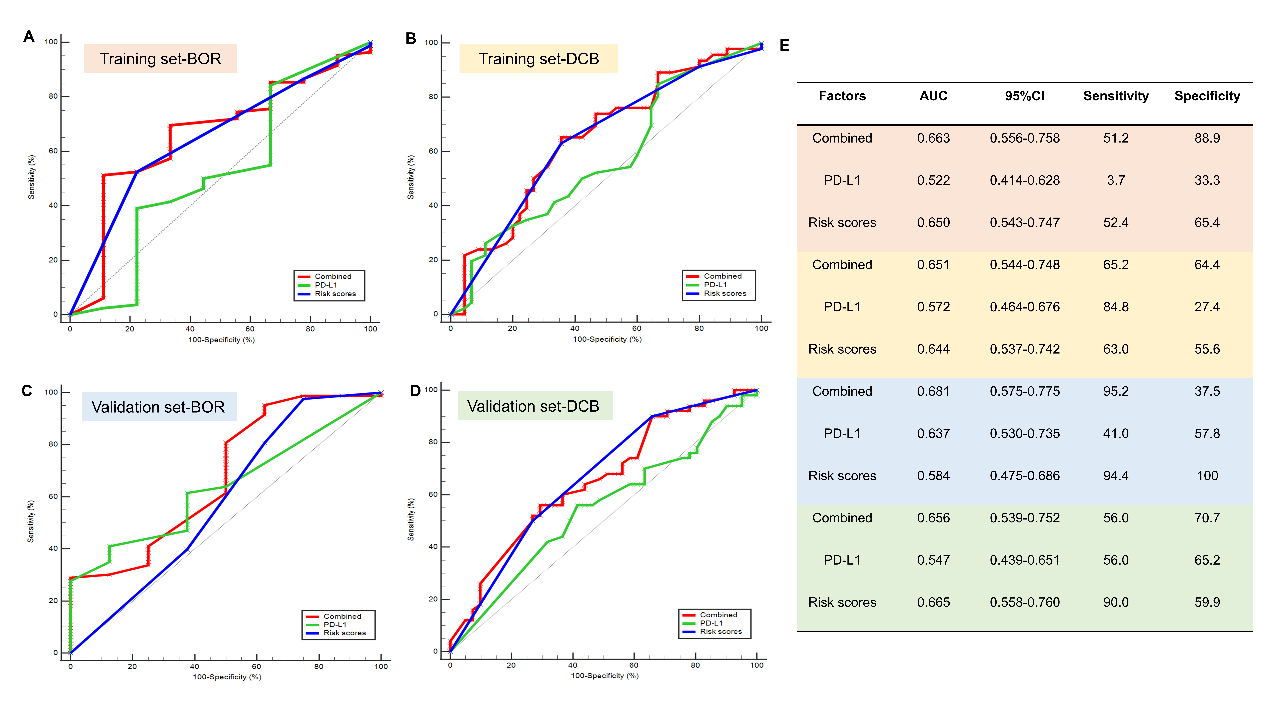


**Fig. S5**

ROC curves of PD-L1 expression, risk score, and the combined factors in predicting (**A**, **C**) best overall response (BOR vs. non-BOR) and (**B**, **D**) durable clinical benefit (DCB vs. NDB) in (**A**-**B**) training cohort and (**C**-**D**) validation cohort. (**E**) presented the AUC, 95%CI, sensitivity, and specificity. ROC receiver operating characteristic, PD-L1 programmed death ligand 1, BOR best overall response, DCB durable clinical benefit, NDB no durable benefit, AUC area under the curve, CI confidence interval.


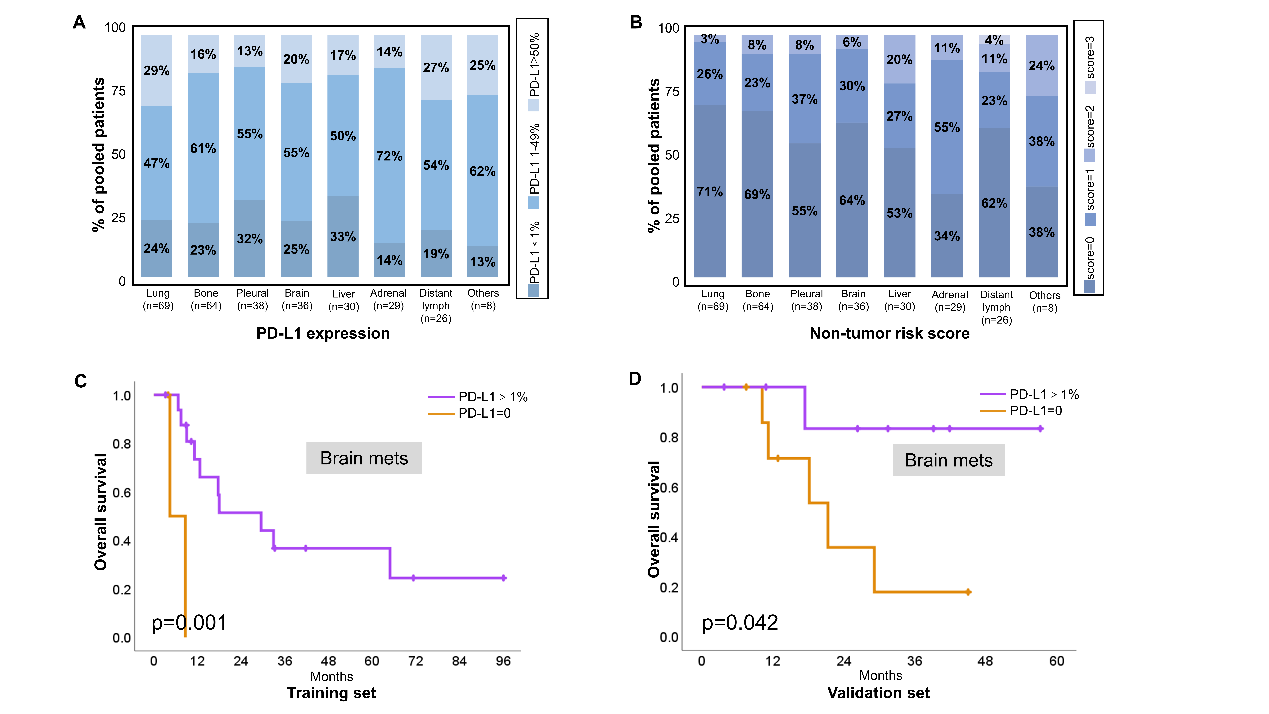


**Fig. S6**

Pooled results in PD-L1 stratification and NTRS with 182 NSCLC patients. Number of patients with different metastases site in (**A**) PD-L1＜1%, 1-49%, and ≧ 50% and (**B**) NTRS (score=0, 1, 2, 3). In the (**C**) training set and (**D**) validation set, the OS was performed among brain metastasis patients between PD-L1＞1% and PD-L1 negative. PD-L1 programmed death ligand 1, NTRS non-tumor related score, NSCLC non-small cell lung cancer, OS overall survival.


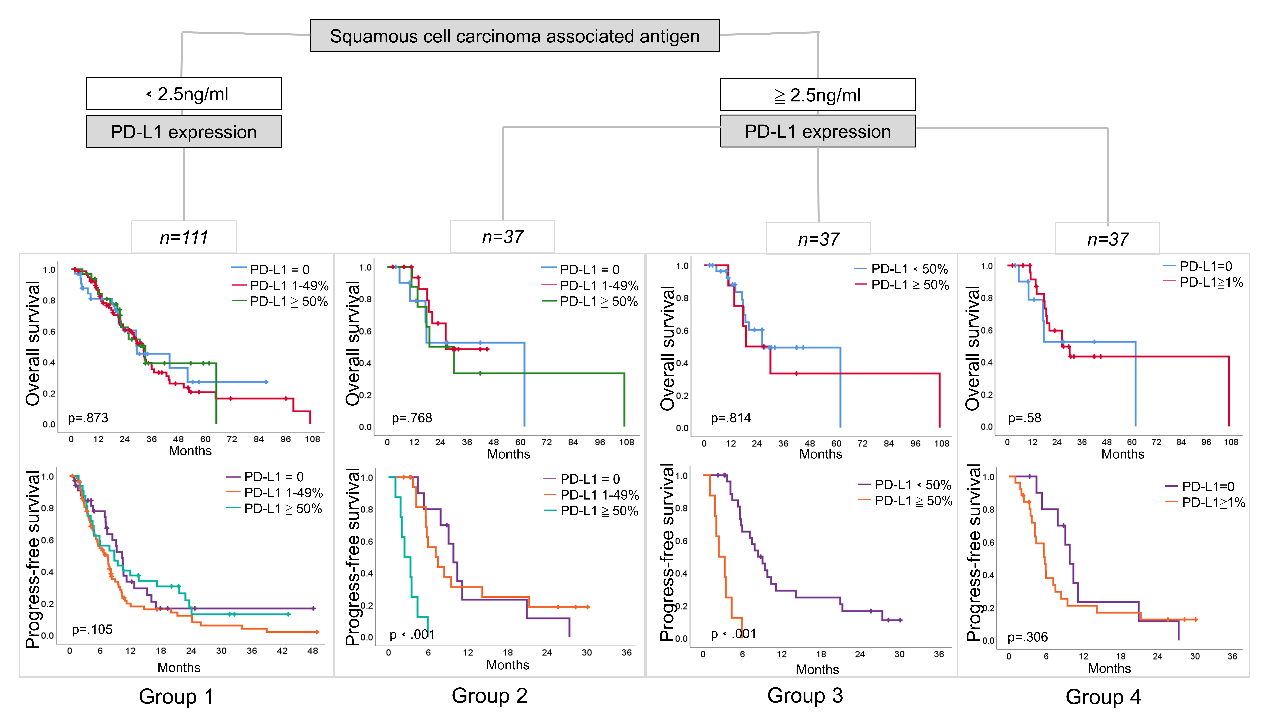


**Fig. S7**

A Kaplan-Meier curve model comparing the hierarchical association of prediction factors associated with OS and PFS among patients with SCC. SCC squamous cell carcinoma antigen. OS overall survival, PFS progress-free survival, SCC squamous epithelial cell carcinoma-associated antigens.


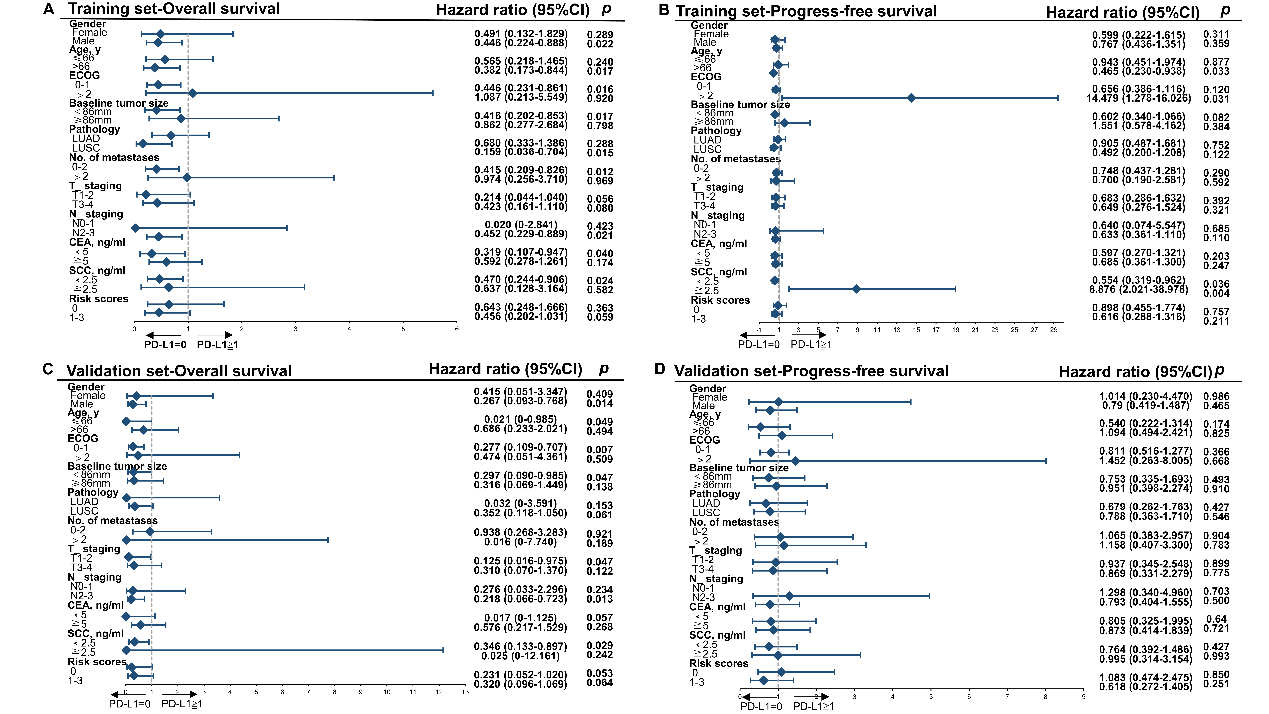


**Fig. S8**

Subgroup analysis using univariate Cox regression was performed to assess the ability of PD-L1 expression to discriminate (**A**, **C**) OS and (**B**, **D**) PFS in patients with different clinical characteristics in (**A**-**B**) training cohort and (**C**-**D**) validation cohort. OS overall survival, PFS progress-free survival, CI confidence intervals, ECOG Eastern Oncology Collaborative Group, TKI tyrosine kinase inhibitors, LUAD Lung adenocarcinoma, LUSC Lung squamous cell carcinoma, CEA carcinoma embryonic antigen, SCC squamous cell carcinoma antigen, PD-1 programmed cell death protein 1, TPS tumor proportion score.
