# Supplementary material for: Non-tumor-related prognostic factors for immunotherapy–chemotherapy or immunotherapy alone as first-line in advanced non-small cell lung cancer (NSCLC)
Source: Clin Exp Med. 2024 Mar 15;24(1):52. doi: 10.1007/s10238-024-01298-z (PMC10942875; doi:10.1007/s10238-024-01298-z)
Supplement: Supplementary file 2 — Supplementary file2 (DOCX 26 kb) [file 10238_2024_1298_MOESM2_ESM.docx]

Table S1 Univariate and multivariate analyses for predictors of overall survival and progress-free survival in the validation cohort.

| Variables | Overall survival | | | | Progress-free survival | | | |
| --- | --- | --- | --- | --- | --- | --- | --- | --- |
|  | Univariate analysis | | Multivariate analysis | | Univariate analysis | | Multivariate analysis | |
|  | HR (95%CI) | *p* | HR (95%CI) | *p* | HR (95%CI) | *p* | HR (95%CI) | *p* |
| Age | 1.014  (0.978-1.052) | 0.440 |  |  | 0.987  (0.959-1.015) | 0.365 |  |  |
| Male | 1.394  (0.730-2.664) | 0.314 |  |  | 1.156  (0.681-1.964) | 0.592 |  |  |
| Smoking | 1.169  (0.635-2.150) | 0.616 |  |  | 0.862  (0.524-1.419) | 0.559 |  |  |
| ECOG | 1.421  (0.564-3.585) | 0.456 |  |  | 1.190  (0.606-2.338) | 0.614 |  |  |
| PD-L1 | 0.705  (0.489-1.016) | 0.051 | 0.656  (0.447-0.962) | 0.031 | 1.004  (0.747-1.350) | 0.978 |  |  |
| [Pathology](javascript:;) | 1.435  (0.832-2.474) | 0.194 |  |  | 1.045  (0.694-1.575) | 0.832 |  |  |
| Maximum diameter | 0.928  (0.852-1.012) | 0.092 |  |  | 0.986  (0.923-1.054) | 0.676 |  |  |
| T_ staging | 0.880  (0.662-1.171) | 0.381 |  |  | 1.014  (0.809-1.271) | 0.905 |  |  |
| N_ staging | 1.082  (0.798-1.467) | 0.611 |  |  | 1.219  (0.941-1.577) | 0.133 |  |  |
| Number of metastases | | | | | | | | |
| 0 vs. 1-2 | 1.809  (0.790-4.140) | 0.161 |  |  | 1.005  (0.527-1.918) | 0.988 |  |  |
| 0 vs. ﹥2 | 0.920  (0.527-1.606) | 0.768 |  |  | 0.951  (0.652-1.387) | 0.794 |  |  |
| 1-2 vs.﹥2 | 0.558  (0.245-1.271) | 0.165 |  |  | 0.768  (0.441-1.339) | 0.353 |  |  |
| LDH levels | 1.002  (1.000-1.005) | 0.057 | 1.001  (0.997-1.004) | 0.419 | 1.000  (0.998-1.003) | 0.875 |  |  |
| ALP levels | 0.998  (0.993-1.003) | 0.457 |  |  | 1.000  (0.998-1.003) | 0.866 |  |  |
| β2-microglobulin levels | 0.708  (0.473-1.058) | 0.092 |  |  | 0.942  (0.693-1.282) | 0.705 |  |  |
| CEA levels | 1.000  (0.999-1.001) | 0.612 |  |  | 1.001  (1.000-1.002) | 0.083 | 1.001  (1.000-1.002) | 0.066 |
| CYFRA 21-1 levels | 1.011  (1.003-1.018) | 0.005 | 1.004  (0.995-1.013) | 0.321 | 1.004  (0.996-1.011) | 0.337 |  |  |
| SCC levels | 1.029  (1.004-1.053) | 0.020 | 1.010  (0.997-1.004) | 0.350 | 1.038  (1.011-1.065) | 0.006 | 1.034  (1.007-1.062) | 0.014 |
| LDL-C levels | 0.972  (0.690-1.369) | 0.872 |  |  | 1.000  (0.766-1.306) | 0.999 |  |  |
| HDL-C levels | 0.856  (0.431-1.697) | 0.655 |  |  | 1.018  (0.990-1.048) | 0.212 |  |  |
| FFA levels | 1.000  (0.999-1.001) | 0.875 |  |  | 1.000  (0.999-1.001) | 0.472 |  |  |
| PLR | 1.001  (0.999-1.003) | 0.169 |  |  | 1.000  (0.999-1.002) | 0.606 |  |  |
| NLR | 0.976  (0.901-1.057) | 0.547 |  |  | 1.003  (0.967-1.041) | 0.877 |  |  |
| HCT | 1.014  (0.963-1.067) | 0.604 |  |  | 0.990  (0.949-1.033) | 0.635 |  |  |
| Albumin levels | 0.983  (0.920-1.049) | 0.601 |  |  | 0.997  (0.946-1.050) | 0.900 |  |  |
| Fibrinogen quantification | 1.001  (0.999-1.003) | 0.412 |  |  | 1.000  (0.999-1.002) | 0.631 |  |  |
| FARI | 1.035  (0.970-1.105) | 0.302 |  |  | 1.008  (0.961-1.056) | 0.753 |  |  |
| NTRS | 2.060  (1.454-2.919) | ＜0.001 | 2.114  (1.493-2.994) | ＜0.001 | 1.425  (1.080-1.880) | 0.012 | 1.395  (1.051-1.851) | 0.021 |

CI confidence interval, OS overall survival, PFS progression-free survival, IQR interquartile range, ECOG Eastern Cooperative Oncology Group, CEA carcinoembryonic antigen, SCC squamous epithelial cell carcinoma-associated antigens, LDH lactate dehydrogenase, ULN upper limit of normal, ALP alkaline phosphatase, LUAD lung adenocarcinoma, LUSC lung squamous cell carcinoma, BTS the sum of the dimensions of baseline target lesions according to RECIST v1.1 criteria, PD-L1 programmed cell death ligand 1, TPS tumor cell proportion score, NTRS non-tumor related score.

Table S2 Correlation of tumor-related factors with NTRS and PD-L1 expression in the pooled data.

| Factors | PD-L1﹤1% | PD-L1  1-49% | PD-L1  ≧50% | Chi2  p value | Score  =0 | Score  ≧1 | Chi2  p value |
| --- | --- | --- | --- | --- | --- | --- | --- |
|  | Patients no. (%) | | | | | | |
| Sex | | | | .613 |  | | .546 |
| Male | 30(68) | 73(74) | 27(67) |  | 88(72) | 42(71) |  |
| Female | 14(32) | 25(26) | 13(33) |  | 35(28) | 17(29) |  |
| Age | | | | .094 |  | | .148 |
| ﹤66 | 15(34) | 51(53) | 22(52) |  | 63(52) | 26(43) |  |
| ≧66 | 29(66) | 45(47) | 20(48) |  | 58(48) | 35(57) |  |
| ECOG | | | | .218 |  | | .649 |
| 0-2 | 37(84) | 82(84) | 38(95) |  | 106(87) | 52(85) |  |
| ﹥2 | 7(16) | 15(16) | 2(5) |  | 15(13) | 9(15) |  |
| Diagnosis | | | | .675 |  | | .554 |
| LUAD | 25(58) | 53(56) | 25(64) |  | 68(58) | 35(58) |  |
| LUSC | 18(42) | 42(44) | 14(36) |  | 49(42) | 25(42) |  |
| Size (mm) | | | | .787 |  | | .066 |
| ≦86 | 30(68) | 68(72) | 30(75) |  | 89(75) | 38(63) |  |
| ﹥86 | 14(32) | 27(28) | 10(25) |  | 29(25) | 22(37) |  |
| T_staging | | | | .609 |  | | .336 |
| T1-2 | 11(40) | 28(40) | 17(50) |  | 36(62) | 20(38) |  |
| T3-4 | 16(60) | 42(60) | 17(50) |  | 52(38) | 23(62) |  |
| N_staging | | | | .72 |  | | ﹤.001 |
| N0-1 | 5(14) | 17(20) | 6(17) |  | 7(8) | 21(30) |  |
| N2-3 | 32(86) | 70(80) | 29(83) |  | 83(92) | 48(70) |  |
| No. of metastases | | | | .511 |  | | .276 |
| 0 | 8(18) | 13(13) | 6(15) |  | 19(16) | 8(13) |  |
| 1-2 | 24(53) | 62(64) | 28(70) |  | 79(65) | 35(57) |  |
| ﹥2 | 13(29) | 22(20) | 6(15) |  | 23(19) | 18(30) |  |
| CEA (ng/ml) | | | | .684 |  | | .497 |
| ﹤5 | 21(47) | 49(50) | 17(43) |  | 60(50) | 27(44) |  |
| ≧5 | 24(53) | 48(50) | 23(57) |  | 61(50) | 34(56) |  |
| SCC (ng/ml) | | | | .005 |  | | .311 |
| ﹤2.5 | 34(76) | 16(47) | 32(80) |  | 99(82) | 46(75) |  |
| ≧2.5 | 11(24) | 18(53) | 8(20) |  | 22(18) | 15(25) |  |

NTRS non-tumor related score, PD-L1 programmed cell death ligand 1, ECOG Eastern Cooperative Oncology Group, LUAD lung adenocarcinoma, LUSC lung squamous cell carcinoma, CEA carcinoma embryonic antigen, SCC squamous cell carcinoma antigen.
